# Structural and Dynamical Insights into the Membrane-Bound α-Synuclein

**DOI:** 10.1371/journal.pone.0083752

**Published:** 2013-12-20

**Authors:** Neha Jain, Karishma Bhasne, M. Hemaswasthi, Samrat Mukhopadhyay

**Affiliations:** 1 Department of Biological Sciences, Indian Institute of Science Education and Research (IISER), Mohali, India; 2 Department of Chemical Sciences, Indian Institute of Science Education and Research (IISER), Mohali, India; Massachusetts General Hospital and Harvard Medical School, United States of America

## Abstract

Membrane-induced disorder-to-helix transition of α-synuclein, a presynaptic protein, has been implicated in a number of important neuronal functions as well as in the etiology of Parkinson’s disease. In order to obtain structural insights of membrane-bound α-synuclein at the residue-specific resolution, we took advantage of the fact that the protein is devoid of tryptophan and incorporated single tryptophan at various residue positions along the sequence. These tryptophans were used as site-specific markers to characterize the structural and dynamical aspects of α-synuclein on the negatively charged small unilamellar lipid vesicles. An array of site-specific fluorescence readouts, such as the spectral-shift, quenching efficiency and anisotropy, allowed us to discern various features of the conformational rearrangements occurring at different locations of α-synuclein on the lipid membrane. In order to define the spatial localization of various regions of the protein near the membrane surface, we utilized a unique and sensitive indicator, namely, red-edge excitation shift (REES), which originates when a fluorophore is located in a highly ordered micro-environment. The extent of REES observed at different residue positions allowed us to directly identify the residues that are localized at the membrane-water interface comprising a thin (∼ 15 Å) layer of motionally restrained water molecules and enabled us to construct a dynamic hydration map of the protein. The combination of site-specific fluorescence readouts allowed us to unravel the intriguing molecular details of α-synuclein on the lipid membrane in a direct model-free fashion. Additionally, the combination of methodologies described here are capable of distinguishing subtle but important structural alterations of α-synuclein bound to different negatively charged lipids with varied head-group chemistry. We believe that the structural modulations of α-synuclein on the membrane could potentially be related to its physiological functions as well as to the onset of Parkinson’s diseases.

## Introduction

**Figure 1:**
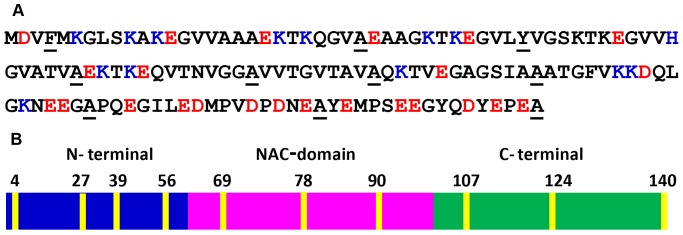
Amino acid sequence and mutational sites of α-synuclein. (A) The amino acid sequence showing negatively (red) and positively (blue) charged amino acids. The mutational sites for incorporating Trp are shown as underscored. (B) Various regions of the protein: N-terminal, NAC-domain and C-terminal. Trp positions are indicated in yellow.

α-Synuclein is a small 140-residue protein that is highly conserved in vertebrates and is preferentially expressed in presynaptic nerve terminals in various regions of the brain [1]–[3]. The precise physiological function of α-synuclein still remains elusive, however, it has been linked with synaptic plasticity [4] and learning [5], vesicle trafficking [6], maintenance of SNARE protein complex [7] and dopamine neurotransmission [8]. Additionally, α-synuclein aggregation is implicated in Parkinson’s disease and in a number of other neurodegenerative disorders [9]–[11]. α-Synuclein belongs to a unique class of proteins known as natively unfolded or Intrinsically Disordered Proteins (IDPs), which has no persistent structure and therefore confronts the conventional sequence-structure-function paradigm [12]–[16]. α-Synuclein consists of three distinct modular domains: (i) positively charged N-terminal region from residues 1–60 has propensity to bind to membranes; (ii) hydrophobic NAC-domain (non-Aβ component of Alzheimer’s disease amyloid) from residues 61–95 is responsible for aggregation [17]; (iii) negatively charged C-terminal region from residues 96–140 is highly flexible and facilitates interaction with Ca^2+^ ion and other molecules (Figure 1A,B) [18]. The N-terminal segment and the NAC-domain have 7 imperfect 11-residue repeats. A remarkable conformational plasticity of this protein-chameleon allows it to adopt a wide range of dynamic structures depending on the environment and binding partners [19]–[22].

Studies using a diverse array of biophysical tools involving circular dichroism (CD) [23]–[25], nuclear magnetic resonance (NMR) [26]–[30], electron paramagnetic resonance (EPR) [31]–[36] and fluorescence [37]–[41] have shown that α-synuclein undergoes a profound conformational transition from a random coil state to α-helical state upon interaction with membrane mimetic such as sodium dodecyl sulfate and anionic phospholipid membranes. The interaction of α-synuclein with lipid membranes is implicated in the physiological roles as well as in the pathological consequences [42]–[46]. Upon interaction with the membrane surface, α-synuclein can adopt two major types of structures, namely, broken horseshoe-type helix [28], [36] and extended helix [36], [47]. Recent single molecule experiments have provided direct and compelling evidence in favor of the two switchable conformational states [48]–[51].

A body of evidence has indicated that the N-terminal segment mediates the membrane binding that is predominantly driven by the electrostatic interactions with the involvement of repeat regions [52], [53]. The relative positioning and the immersion depths of different residues of the polypeptide chain traversing the membranes surface is potentially important for the functional as well as the pathological roles of α-synuclein [42]. Previous results using EPR, fluorescence, neutron reflectometry and computation have provided important molecular details along this line [31]–[55]. In this work we have recorded a variety of environment-responsive site-specific fluorescence observables from a number of single tryptophan variants created along the sequence of α-synuclein (Figure 1A,B) to directly monitor binding-induced folding as well as to unequivocally define the localization of the polypeptide chain on the negatively-charged membrane surface at the residue-specific level. We then utilized a sensitive fluorescence readout of tryptophan, namely, red-edge excitation shift (REES), which originates from a highly ordered micro-environment such as the membrane-water interface comprising a thin (∼15 Å) layer of motionally constrained water molecules [56]-[59]. Results from these experiments allowed us to describe the structural arrangement of α-synuclein bound to the lipid membrane surface. Using the combination of site-specific fluorescence measurements, we have also been able to distinguish the differences in the localization of α-synuclein on a variety of lipid membranes.

## Results

### Residue-specific structural insights into the membrane-bound state

**Figure 2:**
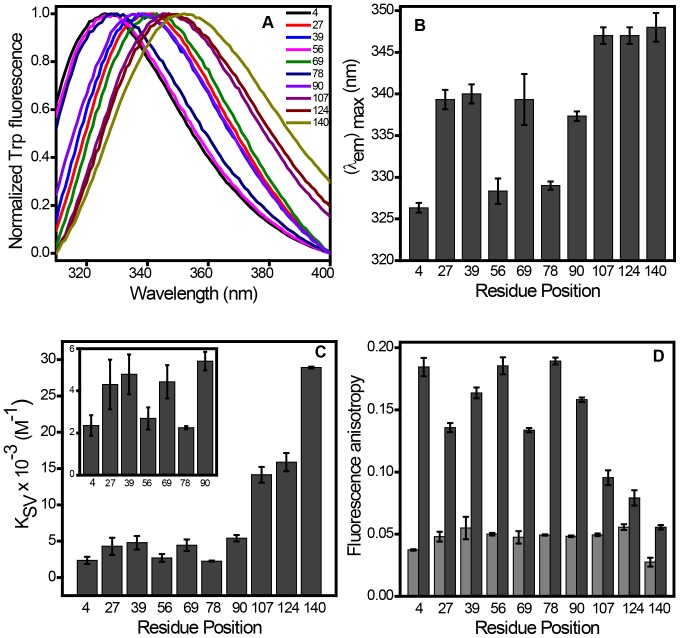
Distinct features of Trp variants located at different residues in the presence of POPG SUVs. (A) Normalized Trp fluorescence spectra showing spectral shift upon lipid binding. (B) Variation in emission maxima along the mutated residues. (C) Accessibility of Trp residues determined by Stern-Volmer constant (*K*_sv_). (D) Fluorescence anisotropy of free IDP state (light grey) and membrane-bound form (dark grey). The standard error was estimated from at least three independent measurements.

We first monitored binding-induced folding behavior of α-synuclein in the presence of small unilamellar vesicles (SUVs) derived from synthetic phospholipids with varied head-group chemistry. Circular dichroism (CD) spectroscopic studies indicated conformational transition from an intrinsically disordered state to a highly helical state upon binding to phospholipid SUVs as previously demonstrated [23]–[25]. Negatively charged SUVs derived from 1-palmitoyl-2-oleoyl-*sn*-glycero-3-phospho (1’-rac-glycerol) (POPG), 1-palmitoyl-2-oleoyl-*sn*-glycero-3-phospho-L-serine (POPS) and 1-palmitoyl-2-oleoyl-*sn*-glycero-3-phosphate (POPA) were able to induce the structural changes under the physiological condition, whereas, no significant change was observed with a zwitterionic neutral lipid such as 1-palmitoyl-2-oleoyl-*sn*-glycero-3-phosphocholine (POPC) (Figure S1). These observations are consistent with the previous reports [23], [24], [40]. In order to monitor structural changes of the polypeptide chain at the residue-specific resolution, we took advantage of the fact that α-synuclein is devoid of any tryptophan (Trp) residue. We generated 10 single-Trp mutants distributed over the polypeptide chain length encompassing N-terminal, NAC and C-terminal domains (Figure 1B). For incorporating Trp, the residue positions at 4, 39, 69, 90, 124 and 140 were chosen based on the earlier reports that demonstrated no alteration in the conformational behavior of α-synuclein [41], [55], [60]. We made additional four single Trp mutants (27, 56, 78 and 107) that retain the capability to switch from a disordered to a helical-state in the presence of lipids (Figure S2). After demonstrating that the incorporation of single Trp does not alter the lipid-binding property of the protein, we monitored a variety of fluorescence readouts that provide region-specific structural information of the protein under free as well as membrane-bound forms. Fluorescence emission spectra for all the Trp residues located at various regions showed a peak at ∼ 348 nm in the free disordered form of the protein suggesting that all these positions are exposed to the bulk water. Upon binding to POPG SUVs, different locations showed a varied extent of blue-shift (λ_em max_ ∼ 327 to 340 nm) indicating the membrane-induced structure formation at the N-terminal segment and the NAC domain, whereas, the Trp residues placed at the C-terminal end did not undergo any detectable blue-shift (λ_em max_ ∼ 348 nm) that is consistent with the fact that predominantly acidic C-terminus does not bind to the membranes (Figure 2A,B) [26], [29], [50], [52]. The residue positions at 4, 56 and 78 showed the maximum blue shift indicating that they are well protected from bulk water, whereas, the positions 27, 39, 69 and 90 remain partially exposed to bulk water. Therefore, the Trp emission maxima provide insights into the positioning of different parts of α-synuclein on the lipid membrane.

In order to gain further insights into the structural arrangement of α-synuclein on the membrane, we next performed acrylamide quenching experiments. In the free monomeric-form of α-synuclein, all of the Trp locations demonstrated high Stern-Volmer quenching constant (*K*_sv_) that corresponds to high bimolecular quenching rate constant (*k*_q_ ∼ 5×10^9 ^M^−1^s^−1^) indicating highly efficient quenching along the polypeptide chain (Figure S3A). On the contrary, the quenching efficiency varied along the Trp residues indicating the variation in the extent of the level of exposure in the lipid-bound state (Figure S3B). The results also indicated that the residue positions 4, 56 and 78 were less accessible to the quencher, whereas the positions 27, 39, 69 and 90 showed a moderate level of exposure to the bulk water (Figure 2C). The C-terminal segment demonstrated complete exposure to the bulk solvent (Figure 2C). Our acrylamide quenching data are in close agreement with a recent report on large unilamellar vesicles bound α-synuclein [55]. Therefore, our quenching data together with emission maxima of Trp indicated varied level of exposure to water as a result of different penetration depths of specific residues of α-synuclein into the membrane.

We next carried out fluorescence polarization experiments, in which the measured steady-state fluorescence anisotropy reports the rotational mobility at a given residue position [61]. The idea behind this experiment is that the binding-induced folding would impart considerable rotational restriction at a given region of the polypeptide chain containing Trp and would result in a sharp increase in the fluorescence anisotropy. We carried out anisotropy experiments for all single-Trp variants of α-synuclein along the sequence to map the dampening in the rotational dynamics from the N- to the C-terminal end. In the free form, anisotropy was low (*r*_ss_ = 0.05±0.01) for all the positions in α-synuclein indicating an intrinsically disordered state without any specific structural propensity (Figure 2D). In the lipid-bound form, the anisotropy values along the sequence showed varied extent of dampening of dynamics as a result of gain in the structure at different regions of the protein (Figure 2D). Based on the measured anisotropy at various residue positions, we constructed a fluorescence anisotropy map, which revealed that the N-terminal end and NAC-domain are tightly bound and structured (*r*_ss_>0.10), whereas, C-terminal end does not seem to participate in the folding event (*r*_ss_<0.10). The high anisotropy values at positions 4, 56 and 78 (*r*_ss_  =  0.18±0.01) are suggestive of restriction in mobility upon binding to the membrane. The positions 27, 39, 69 and 90 demonstrated little lower values of anisotropy indicating somewhat higher degree of flexibility compared to the position 4, 56 and 78. The structural organization progressively diminishes as Trp is moved from the NAC- to the C-terminal region.

Fluorescence spectral shift, quenching and anisotropy experiments together provide residue-specific structural organization of the α-synuclein conferred upon membrane binding. All these measurements indicated the positions 4, 56 and 78 of α-synuclein are structurally highly restrained, buried and not available to bulk exterior water. On the contrary, the positions 27, 39, 69 and 90 are moderately restrained and experience partial exposure to bulk water. However, these measurements do not allow us to directly address the residue-specific localization of the protein on the membrane surface.

### Residue-specific localization of α-synuclein on the membrane surface

**Figure 3:**
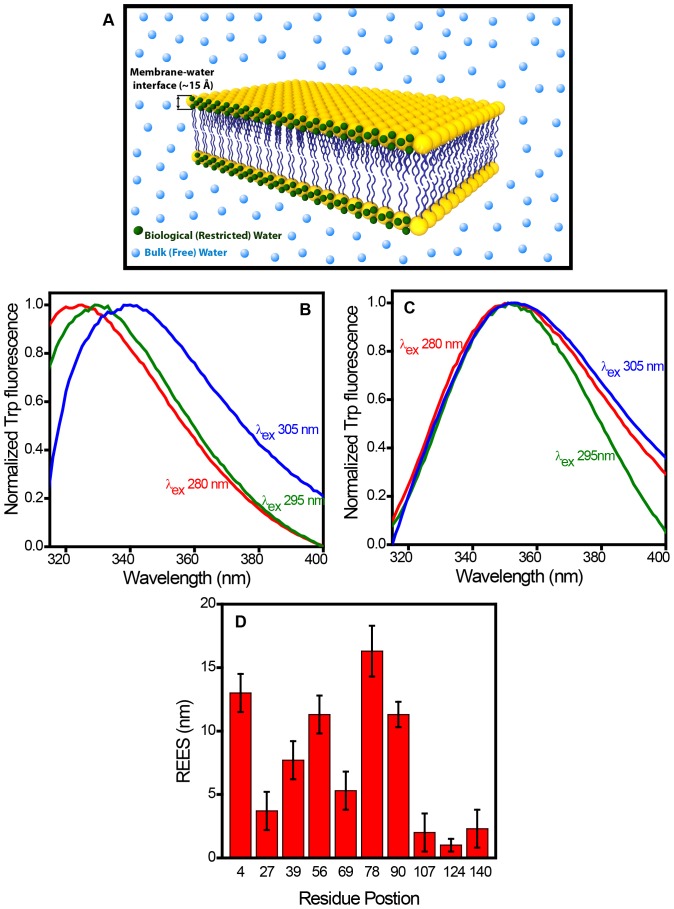
Red-edge excitation shift (REES) of tryptophans in membrane-bound α-synuclein. (A) A cartoon of membrane bilayer depicting bulk (free) and restricted (biological) water. Fluorescence emission spectra of Trp 78 (B) and Trp 140 (C) varying the excitation wavelength (λ_ex_) from 280 nm to 305 nm in the presence of POPG SUVs. (D) REES observed at different residue positions. The standard error was estimated from at least three independent measurements.

We next asked the question: How are the different residues of the protein spatially distributed with respect to the membrane surface? In order to answer this question, we set out to perform experiments that would allow us to distinguish the proximal and distal tryptophans with respect to the membrane surface. Here we took advantage of the fact that the nature of water molecules at the membrane-water interface is distinctly different from that of the bulk water molecules. The membrane interface comprises a thin (∼ 15 Å) layer of motionally restrained water molecules that create highly ordered and viscous micro-environment (Figure 3A) [56]–[59]. We conjectured that the residues that are localized in this thin layer of highly ordered water molecules will report its fluorescence readout that is sensitive to micro-viscosity. One of such fluorescence readouts is the red-edge excitation shift (REES) that represents a unique and sensitive approach to monitor the dynamics of restricted water molecules at the membrane interface [57]. In bulk non-viscous media, fluorescence emission maxima is independent of excitation wavelength since the timescale of water reorientation (hydration dynamics in response to a transiently created excitation dipole) is orders of magnitude faster (picoseconds) compared to the fluorescence decay timescale (nanoseconds). In a viscous micro-environment, such as in membrane-interface, the water reorientation time gets slowed down by orders of magnitude and competes with the fluorescence lifetime, and as a result, the emission maxima exhibits a progressive shift towards the longer wavelength when the excitation wavelength is gradually shifted to the red edge of the absorption band [57]. It has been shown that the REES serves as an extremely reliable indicator of motionally restrained water molecules. Tryptophan fluorescence from proteins has also been shown to demonstrate REES under restricted environment such as membrane-water interface [56], [57], [59].

In the disordered (unbound) state of α-synuclein, Trp residues placed along the sequence showed very little or no REES (∼ 1–2 nm) indicating the absence of organized water molecules. On the contrary, in the membrane-bound form, different residue positions demonstrated a varied extent of REES indicating their proximal and distal positioning with respect to the membrane interface (Figure S4). Figure 3B and 3C show the fluorescence spectra for POPG-bound α-synuclein (Trp 78 and 140, respectively) recorded by exciting at three different wavelengths (280 nm, 295 nm and 305 nm) at the red-edge of the tryptophan absorption maxima. These spectra clearly indicate a progressive shift in the emission maxima for Trp 78 variant as a function of excitation wavelength. Trp 140 located at the end of C-terminus did not undergo any shift. In order to rule out the possibility of structural heterogeneity contributing to the shift [57], we performed a control experiment with Trp 78 variant of the protein. We recorded fluorescence spectra in the absence and in the presence of a quencher (potassium iodide) and observed that the spectral shape and emission maxima remain unaltered (Figure S5). Therefore, the REES that we observe is indeed due to slow water reorientation dynamics around Trp and not due to any structural heterogeneity. We then plotted the magnitude of REES (in nm) for each residue position in the membrane-bound form to construct a dynamic hydration map that depicts the regions that are localized within the ordered layer of motionally restrained water molecules at the membrane interface (Figure 3D). The initial part of the N-terminal segment (Trp 4) demonstrated a large extent of the REES (∼ 13 nm), whereas, the middle region (Trp 27 and 39) exhibited much lower REES (∼ 4 nm and ∼ 7 nm, respectively). The end of the N-terminal part (Trp 56) and the NAC-domain residues 78 and 90 showed significantly high REES (∼ 11 nm, 16 nm and 11 nm, respectively), whereas, the initial part of NAC-domain (Trp 69) exhibited much lower value REES (∼ 5 nm). The C-terminal residues did not show significant REES (∼ 2-3 nm).

**Figure 4:**
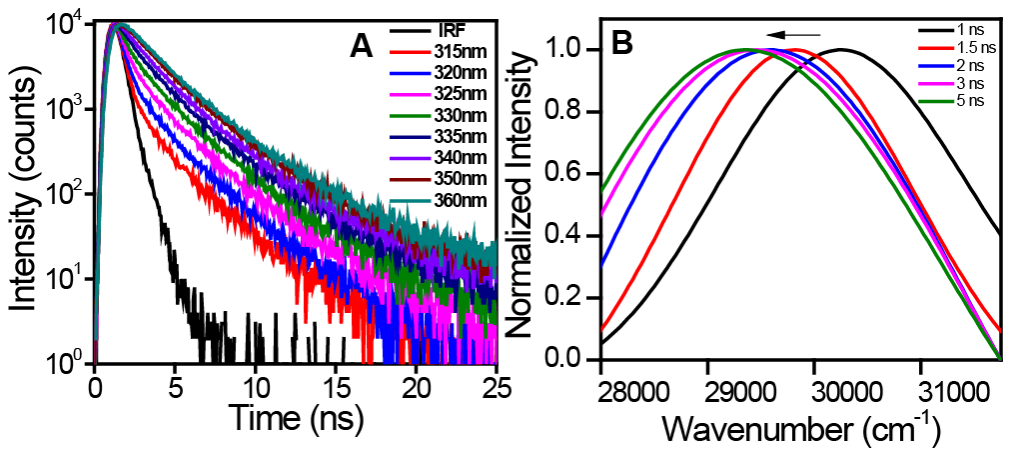
Time-resolved fluorescence studies to monitor ultraslow hydration dynamics. (A) The nanosecond time-resolved decay of Trp 78 of α-synuclein in the presence of POPG SUVs showing progressive increase in the lifetime as a function of emission wavelength (IRF: instrument response time). (B) Time-resolved emission spectra showing gradual red-shift (in cm^−1^) on the nanosecond timescale.

The REES arises due to the rate of water reorientation dynamics that is either comparable to or slower than the fluorescence lifetime. In order to directly monitor the water dynamics, we next embarked upon studying the timescale of hydration dynamics of α-synuclein in the presence of POPG SUVs using time-resolved emission spectra (TRES) that measures the time-dependent Stokes shift (TDSS) on the nanosecond timescale. In order to perform TRES experiments, we chose Trp 78 variant which lies in the NAC-domain and demonstrates the maximum extent of REES. Figure 4A shows the time-resolved decay of Trp fluorescence monitored at different emission wavelength. The fluorescence decays become progressively longer with an increase in the average fluorescence lifetime as a function of emission wavelength and provide a clear signature of slow solvent relaxation in the excited state. The TRES constructed from the decay analysis exhibited TDSS on the nanosecond timescale (Figure 4B). These results provide compelling evidence in favor of ultraslow water relaxation arising out of localization of the Trp-residue at membrane-interface comprising a highly ordered water layer with a constrained mobility.

The REES is a direct measure of the residues getting embedded into the interfacial region of the membrane comprising restrained water molecules that are dynamically distinct from the bulk water molecules. These results are also in line with the spectral shift, quenching and anisotropy observed for different residue positions of membrane-bound α-synuclein. Although different fluorescence readouts report different structural aspects of the protein on the membrane (spectral-shift: environment; quenching efficiency: solvent accessibility; anisotropy: rotational mobility; REES: solvent relaxation). These structural attributes seem to correlate well with each other. Therefore, utilizing all four readouts (spectral-shift, quenching efficiency, anisotropy and REES) together represent a fairly comprehensive structural description of membrane-bound α-synuclein. Next, we embarked upon utilizing this combination of readouts to delineate the structural differences for α-synuclein bound to a variety of negatively charged membranes.

### Influence of the lipid head-group chemistry on the structural arrangement of α-synuclein

**Figure 5:**
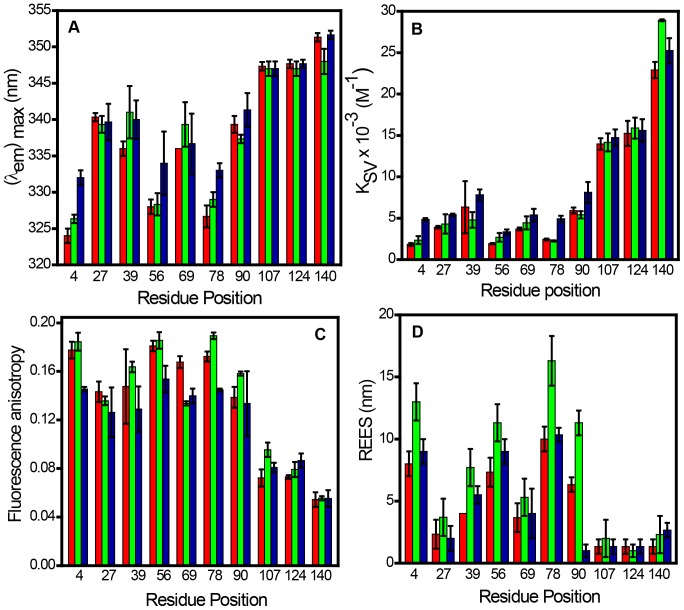
Fluorescence readouts of different Trp residues in the presence of SUVs derived from POPA (red), POPG (green) and POPS (Blue). Changes in (A) emission maxima (B) Stern-Volmer constants (C) Fluorescence anisotropy (D) REES. The standard error was estimated from at least three independent measurements.

Figure 5 shows the plot of all four fluorescence readouts (spectral shift, quenching efficiency, anisotropy and REES) of α-synuclein in the presence of SUVs prepared using negatively charged lipids namely POPA, POPG and POPS. These results indicated that the overall pattern of these readouts remains same for all the lipids. However, a closer inspection of these values reveals that there are small but measurable structural differences of α-synuclein in different lipids. For instance, the residue position 4 exhibited more blue-shift in the presence of POPA and more red-shift in the presence of POPS when compared with the emission maxima observed for POPG (Figure 5A). The spectral shift indicated that the residue location 4 is little more inserted into the hydrocarbon region of POPA lipid whereas the reverse is found for POPS lipid in which the residue is slightly more exposed to bulk water. This finding also corroborated the quenching results (Figure 5B). Additionally, the anisotropy plot demonstrated a similar trend indicating Trp 4 is motionally restricted in both POPG and POPA lipids but remains relatively flexible in POPS presumably because of higher exposure to bulk water (Figure 5C). Interestingly, Trp 4 in both POPA and POPS exhibited lower REES compared to that is observed for POPG (Figure 5D). These differences also are observed for the other positions such as 56 and 78 that are strongly bound to the membrane but are less pronounced for the positions such as 27, 39, 69 and 90 that demonstrate weak to moderate incorporation into the membrane surface. On the contrary, the C-terminal locations did not show any significant changes in the fluorescence readouts in the presence of different lipids. No significant interaction was observed for a zwitterionic lipid POPC as demonstrated by a small spectral-shift, high quenching efficiency, low anisotropy and insignificant REES for all the Trp variants. Our results on POPC are also in good agreement with the previous reports [23], [24], [40] (Figure S6). These results indicate that the N-terminal region gets immersed more into the POPA membrane compared to POPG membrane resulting in more inward displacement of the residues from the ordered membrane-water interface. In contrast, the N-terminal end penetrates less efficiently into the POPS membrane compared to POPG membrane conceding more outward displacement of the residues from the interface. Taken together, these results distinguish subtle but important structural features of membrane-bound α-synuclein with varied lipid head-group chemistry.

## Discussion

α-Synuclein is known to possess an astonishing conformational plasticity and undergoes a large-scale disordered-to-helix conformational transition in the presence of lipid membranes. Using our single Trp mutants generated along the sequence of α-synuclein from the N- to the C-termini, we have been able to gain structural and dynamical insights at specific locations of the protein on the membrane surface. The selective insight from each of these residues is independent of the other part of the chain and allows us to map out the structural features of the protein on the lipid membrane. The spectral-shift of Trp is a reliable indicator of the micro-environment and the quenching efficiency is a measure of the level of exposure to the exterior aqueous environment. These data allow us to conclude that the residue locations 4, 56 and 78 are highly buried and 27, 39, 69 and 90 are moderately buried among the residues we studied. The fluorescence anisotropy is a measure of the probe mobility and indicated rotational hindrance as a result of strong lipid-protein interactions at locations 4, 56 and 78. The anisotropy data also showed relatively weaker interactions at locations 27, 39, 69 and 90. The chain progressively starts emanating out of the membrane surface and the C-terminal residues do not localize themselves anywhere near the membrane surface. Even in the membrane-bound form, C-terminal end behaves like a disordered chain, an observation that is consistent with previous reports [26], [29], [50], [52]. Although the anisotropy map depicted in Figure 2D provides important structural insights at the residue-specific level, it lacks the information about the depth-profile of the α-synuclein chain on the membrane. In order to obtain the precise localization of different residues near the membrane surface, we utilized a reliable indicator, such as REES, that reports the hydration dynamics around a fluorophore. This type of highly ordered (interfacial) water, also termed as ‘biological water’, has been conjectured to play a pivotal role in a variety of crucial biomolecular processes [62], [63]. The extent of REES depends on whether or not the residues are localized in the outer leaflet of the bilayer comprising ordered water layer. Therefore, our REES studies report on otherwise ‘optically silent’ water molecules that are important to characterize the spatial localization of various residue positions of α-synuclein traversing the membrane surface. Our nanosecond time-resolved fluorescence experiments confirmed the ultraslow hydration dynamics as the origin of REES in the membrane-bound form of α-synuclein.

Our results from site-specific fluorescence measurements elucidate the intriguing molecular details of the binding mode of α-synuclein to the negatively charged membrane surface. The structural features are largely consistent with the helical wheel model proposed based on EPR experiments that illustrates the localization of different residue positions arising from the helical turn of α-synuclein on the lipid membrane [31], [35]. We would like to point out that our results are based on an ‘intrinsic’ Trp probe that is a natural amino acid residue as opposed to an ‘extrinsic’ unnatural spin label used in the EPR experiments. There are some minor deviations from the proposed helical wheel model, especially at locations 39 and 78. Our results corroborate the recent finding that indicated the residue 39 experiences more polar environment and therefore tends to deviate from the perfect helical wheel model [55]. Additionally, our experiments revealed that the position 78 is well penetrated into the lipid head-group region containing bound water and demonstrates the maximum extent of REES. Our findings, consistent with the conclusions of the recent work [55], suggest that there could potentially be curved helix, which can cause some of the residue positions becoming more water- or lipid-exposed than that is predicted by the typical helical wheel model. Additionally, there could be a coexistence of long contiguous helix and horse-shoe type broken helix, which can introduce local flexibility [47].

The other important feature of our work is that the combination of fluorescence readouts presented here are capable of distinguishing subtle but important structural differences of α-synuclein bound to different negatively charged lipids. The highest value of REES observed for POPG suggested the immersion of α-synuclein into the outer leaflet of the membrane containing ordered water layer. Interestingly for POPA, a relatively low extent of REES, but significant blue-shift, low quenching efficiency and high anisotropy reveal that α-synuclein penetrate further inside into the hydrophobic region of the bilayer. In sharp contrast to POPG and POPA, POPS harbors α-synuclein on the outer surface where the protein is partially exposed to the bulk water indicated by lower values of shift, anisotropy and REES.

In summary, our results on site-specific REES in combination with the other spectroscopic signatures provide molecular underpinnings of α-synuclein folding on the lipid membranes in a direct model-free fashion. The residues embedded into the membrane may alter the surface topology and lipid packing. These structural modulations could potentially be related to the fusion propensity of presynaptic vesicles. Additionally, the polypeptide segments that protrude from the membrane surface can promote protein-protein interactions and serve as initiators in α-synuclein aggregation. This work highlights the importance of studying site-specific hydration dynamics of proteins in order to decipher the structure and organization of lipid-protein complexes. Our results also demonstrates that the structural arrangement of α-synuclein is slightly altered depending on the head-group chemistry of phospholipids that can potentially be of prime relevance for its function as well as for the onset of Parkinson’s diseases. We believe that the combination of the methodologies described here will find broad applications in the study of lipid-protein interactions for a variety of other membrane-associated proteins implicated in physiological functions as well as in disease progression.

## Materials and Methods

### Materials

Lipids (POPC, POPS, POPA and POPG) were purchased from Avanti Polar Lipids (Alabaster, AL) and all other chemicals were purchased from Sigma (St. Louis, MO) and used as received.

### Mutagenesis, expression and purification

Human α-synuclein cloned in pT7-7 plasmid and transformed into *E.coli* BL21(DE3) was used for protein expression. Single Trp was introduced at ten different residues positions (F4W, A27W, Y39W, A56W, A69W, A78W, A90W, A107W, A124W, and A140W) using site-directed mutagenesis kit, QuikChange, purchased from Stratagene. The primer sequences for site-directed mutagenesis are given in Table S1. All the mutants were confirmed by DNA sequencing. The recombinantly expressed protein was purified using the protocol described previously [64] with minor modifications. The overnight grown culture was transferred to fresh media and induced with 0.8 mM IPTG for 4h. The cell pellets were harvested by centrifuging at 4000 rpm for 30 min and then resuspended in the lysis buffer (10 mM Tris, 150 mM NaCl, 1 mM EDTA pH 8). The lysed cells were then boiled at ∼ 90 °C for 20 min and then centrifuged at 11,000 rpm for 20 min to separate the cell debris. To precipitate the DNA, 10% streptomycin sulphate (136 µL/mL) and glacial acetic acid (228 µL/mL) were added to the supernatant and centrifuged at 11,000 rpm. The protein was preferentially precipitated using equal volume of saturated ammonium sulphate solution. The precipitated protein was further subjected to ethanol and ammonium acetate precipitation followed by re-suspension of the pellet in 10 mM Tris pH 7.4. The resultant solution was then loaded onto a Q-Sepharose anion exchange column and the protein was eluted at ∼ 300 mM NaCl. The fractions containing pure α-synuclein were pooled and dialyzed against the buffer (10 mM HEPES and 50 mM NaCl pH 7.4). The concentration of wild-type protein was determined using ε_275_  =  5,600 M^−1^cm^−1^ [64] and for all the mutants (except for Y39W) was determined using ε_280_ = 10,810 M^−1^cm^−1^ and ε_280_ = 9,970 M^−1^cm^−1^ (Y39W) [41]. The purified protein was concentrated to ∼ 200 µM and stored at -80°C. Prior to every experiment, all the protein solutions were filtered through 50 kDa AMICON to remove oligomers, if any, and then concentrated using 3 kDa AMICON (Millipore). The stock of proteins was diluted to get a final concentration of 50 µM.

### Preparation of small unilamellar vesicles (SUVs)

Lipid vesicles (SUVs) were prepared using previously reported procedure with slight modifications [33]. Briefly, an appropriate amount of chloroform solution of lipid was taken in a round bottom flask and purged with a gentle stream of nitrogen followed by vacuum desiccation for two hours to ensure complete removal of the organic solvent. The dried lipid film was then hydrated in DPBS (Dulbecco’s phosphate buffer saline: 2.67 mM KCl, 1.47 mM KH_2_PO_4_, 138 mM NaCl, and 8.06 mM Na_2_HPO_4_; pH 7.4) buffer to obtain a final lipid concentration of 10 mM. Hydration was carried out for one hour with intermittent vortexing. Liposomes were then subjected to five freeze-thaw cycles alternating between liquid nitrogen and water bath (preset at 42°C) for one minute each followed by sonication in a bath sonicator for one hour to obtain homogenous solution of SUVs. The size of SUVs was determined using DAWN 8 Helios MALS system (Wyatt Technology). The average hydrodynamic radius of SUVs was found to be ∼ 30 nm. Vesicles were prepared fresh for each set of experiments. 10 mM stock of liposomes was diluted into the protein to get a final concentration of 2 mM. For all our Trp fluorescence experiments, we have used a lipid to protein ratio of 40 1.

### CD experiments

Far UV CD spectra for all Trp variants were measured in the absence and in the presence of SUVs derived from different lipids, using the Chirascan CD spectrophotometer (Applied Photophysics, Leatherhead, UK). The stock of proteins and liposomes were diluted in DPBS buffer to get a final concentration of 25 µM and 1 mM, respectively. The spectra were recorded in 1 mm pathlength cuvette with a scan range of 200–260 nm. All the spectra were averaged over 3 scans. The scans were buffer subtracted and smoothened using Pro-Data software provided with the instrument and plotted using Origin software.

### Steady-state fluorescence measurements

The steady-state fluorescence measurements were recorded on Fluoromax-4 (Horiba Jobin Yvon, NJ). The final concentration of α-synuclein was 50 μM for all the fluorescence measurements. For measuring tryptophan fluorescence, the samples were excited at 295 nm and the emission spectra were background subtracted and corrected. The quenching experiments were performed using acrylamide as quencher. The Stern-Volmer quenching constant (*K*_sv_) was determined using the following relationship [61]:

*F*_0_/*F*  =  1 + *K*_sv_[*Q*] (1)

where, F_0_ and F are the tryptophan fluorescence intensities in the absence and presence of quencher.

The steady-state fluorescence anisotropy (*r_ss_*) is given by the following relationship [61]:

*r_ss_*  =  (*I*_||_ – *I*_⊥_ *G*)/(*I*_||_ + 2*I*_⊥_ *G*) (2)

where *I*_||_ and *I*_⊥_ are fluorescence intensities collected using parallel and perpendicular geometry, respectively, with respect to the excitation polarizer. The perpendicular components were corrected using a G-factor. The standard error was estimated from at least three independent measurements.

### REES measurements

For REES measurements, the excitation wavelength was varied between 280 and 305 nm and the emission scan range was from 310 to 400 nm. The excitation bandpass was adjusted to 0.5 nm (for 280 and 295 nm) and 1 nm (for 305 nm). The emission bandpass was fixed to 3 nm. The spectra were averaged over 3 scans except for excitation with 305 nm where 5 scans were collected for each spectrum. All the spectra were background subtracted, corrected and plotted using Origin software. The standard error was estimated from at least three independent measurements.

### Time-resolved emission spectra (TRES) measurements

For the TRES measurements, the time-resolved fluorescence decays of the samples were collected using a TCSPC setup (Fluorocube, Horiba Jobin Yvon, NJ). Aqueous solution of ludox was used to record the instrument response function (IRF), which was measured to be ∼ 1 ns. In order to obtain a good signal-to-noise ratio, 10,000 counts were collected at the peak. For these measurements, 295 nm LED was used as an excitation source and emission decays were collected at a magic angle (54.7°) in the wavelength range from 315–360 nm with an interval of 5 nm and a bandpass of 12 nm. The intensity decays were deconvoluted and analyzed as a sum of exponentials. The fits were used to construct the TRES using normalization with respect to the steady-state fluorescence intensity that was obtained from the steady-state fluorescence spectrum. The spectra were then normalized and fitted to log normal function using Origin software.

## Supporting Information

Figure S1 **CD spectra of wt α-synuclein in the free and in the lipid-bound state.**
(PDF)

Figure S2 **CD spectra of wild-type and tryptophan mutant of α-synuclein in the absence (black) and in the presence (red) of POPG SUVs showing the structural transition from the disordered to the helical state.** The CD ratio plot ([θ]_222_/[θ]_205_) indicates that all of the variants undergo conformational transition nearly to the same extent.
(PDF)

Figure S3 **Bimolecular quenching constant (** ***k_q_*** **) of tryptophan at different residue positions of α-synuclein in the absence (A) and in the presence (B) of POPG SUVs.**
(PDF)

Figure S4 **Red-edge excitation shift (REES) of tryptophans located at different residue positions in α-synuclein in the presence of POPG SUVs.** All the fluorescence spectra were normalized to show the shift upon changing the excitation wavelength from 280 nm to 305 nm (280 nm ex: black; 295 nm ex: red; 305 nm ex: blue).
(PDF)

Figure S5 (A) Fluorescence spectra of Trp 78 of α-synuclein in the presence of POPG SUVs in absence (red) and in the presence (black) of 0.2 M potassium iodide (excited at 295 nm). (B) Normalized spectra showing no shift and no change in the spectral shape in the absence and in the presence of potassium iodide indicating that the observed shifts in the REES experiments are indeed due to slow water relaxation around Trp in the excited state.
(PDF)

Figure S6 **Different readouts of Trp variants in the presence of POPC SUVs.** (A) Emission maxima (B) Fluorescence anisotropy (C) REES (D) Stern-Volmer constants.
(PDF)

Table S1 **Primer sequences for site-directed mutagenesis.**
(PDF)
