# Supplementary figures and images for: Structural and Dynamical Insights into the Membrane-Bound α-Synuclein
Source: PLoS One. 2013 Dec 20;8(12):e83752. doi: 10.1371/journal.pone.0083752 (PMC3869795; doi:10.1371/journal.pone.0083752)

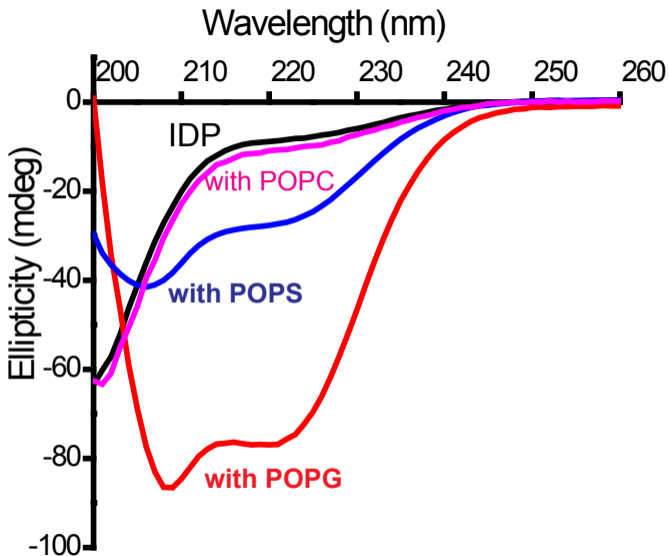

Supplement: Figure S1 — CD spectra of wt α-synuclein in the free and in the lipid-bound state. (PDF) [file pone.0083752.s001.pdf]

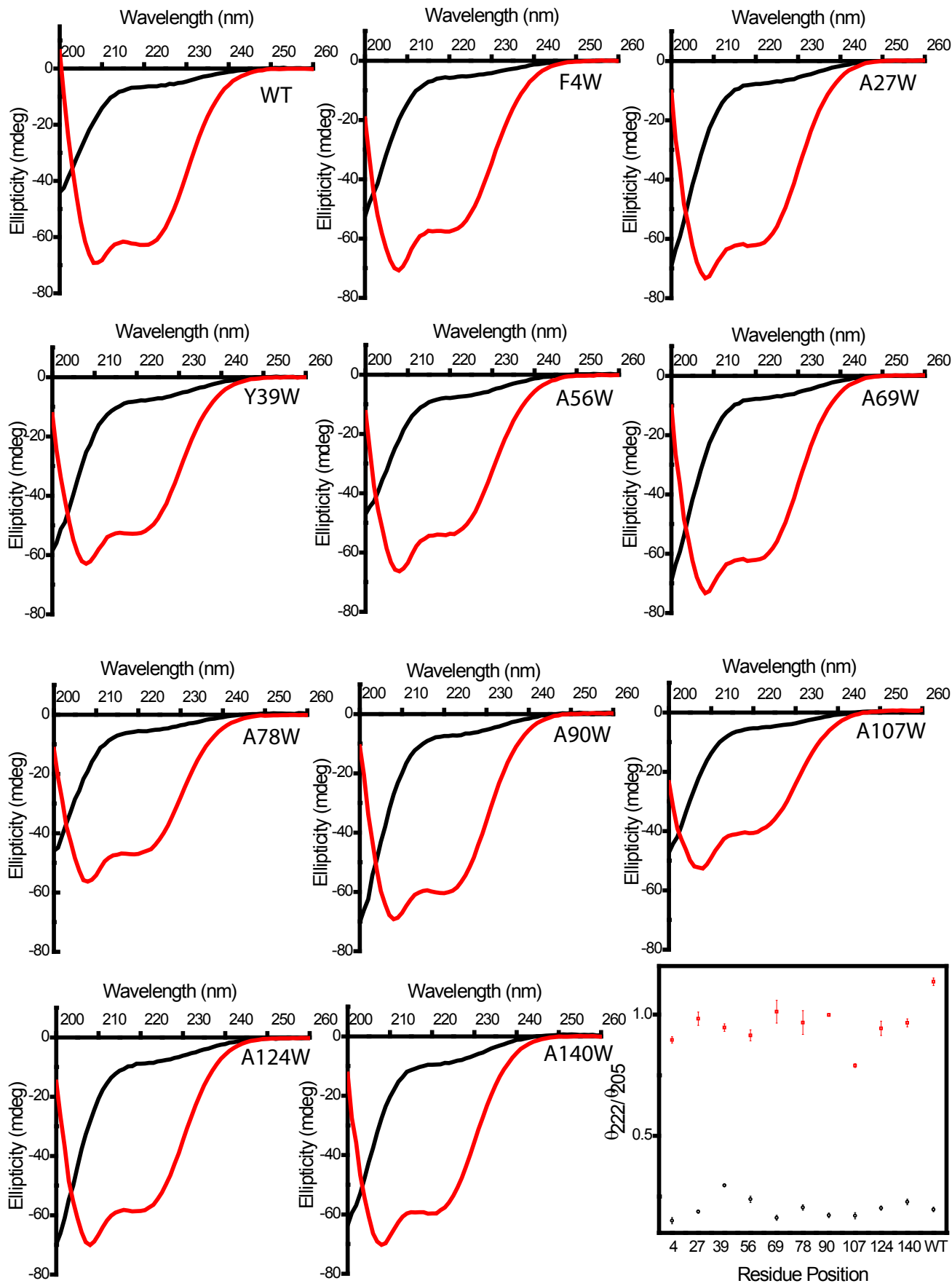

Supplement: Figure S2 — CD spectra of wild-type and tryptophan mutant of α-synuclein in the absence (black) and in the presence (red) of POPG SUVs showing the structural transition from the disordered to the helical state. The CD ratio plot ([θ]222/[θ]205) indicates that all of the variants undergo conformational transition nearly to the same extent. (PDF) [file pone.0083752.s002.pdf]

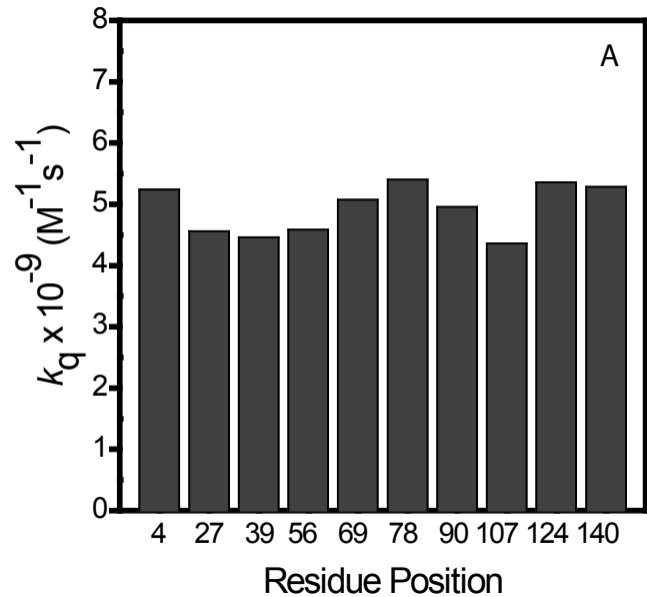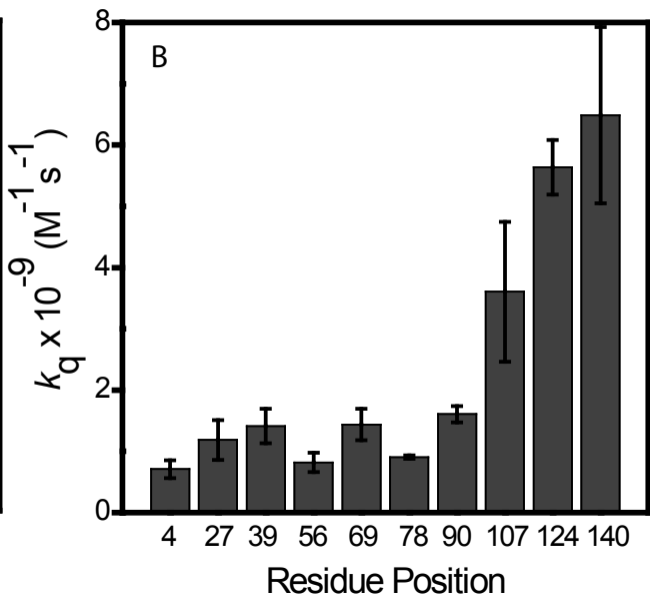

Supplement: Figure S3 — Bimolecular quenching constant ( kq ) of tryptophan at different residue positions of α-synuclein in the absence (A) and in the presence (B) of POPG SUVs. (PDF) [file pone.0083752.s003.pdf]

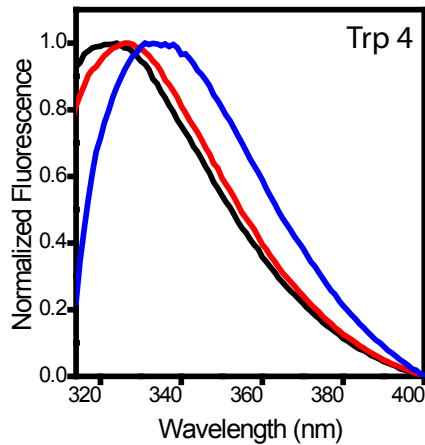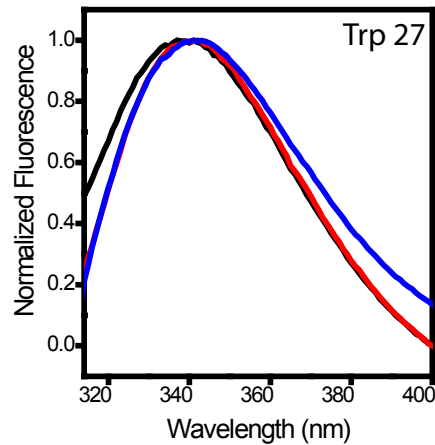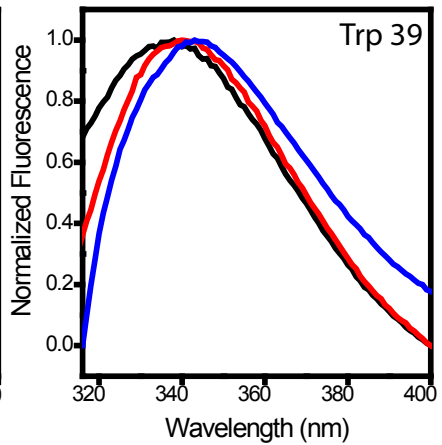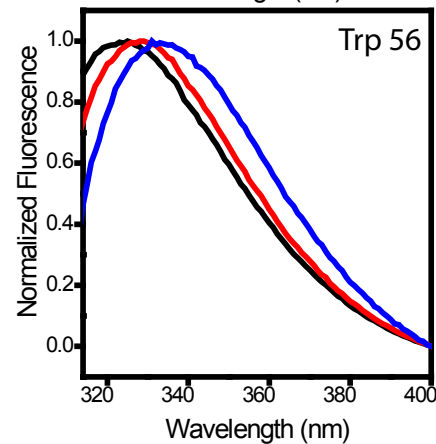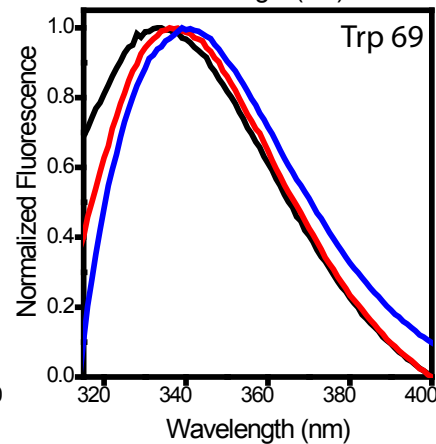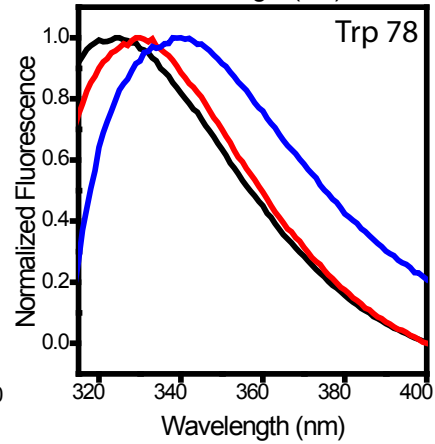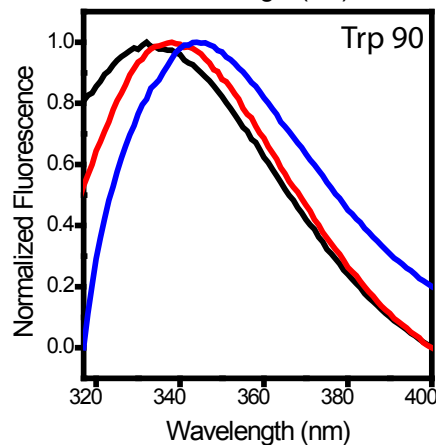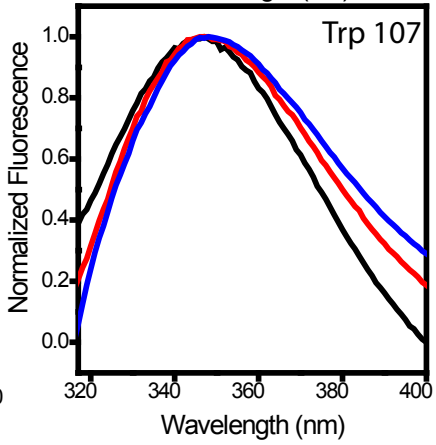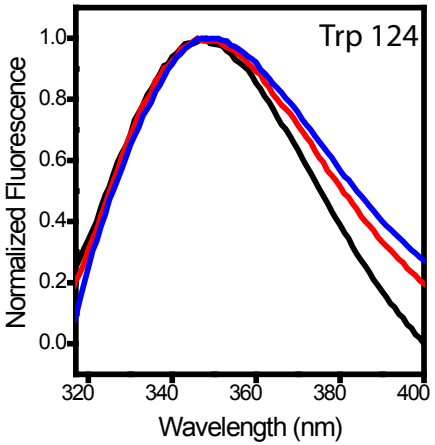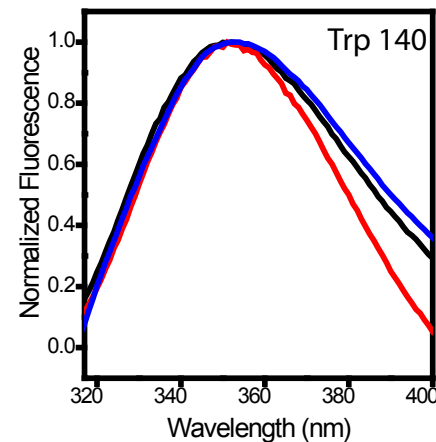

Supplement: Figure S4 — Red-edge excitation shift (REES) of tryptophans located at different residue positions in α-synuclein in the presence of POPG SUVs. All the fluorescence spectra were normalized to show the shift upon changing the excitation wavelength from 280 nm to 305 nm (280 nm ex: black; 295 nm ex: red; 305 nm ex: blue). (PDF) [file pone.0083752.s004.pdf]

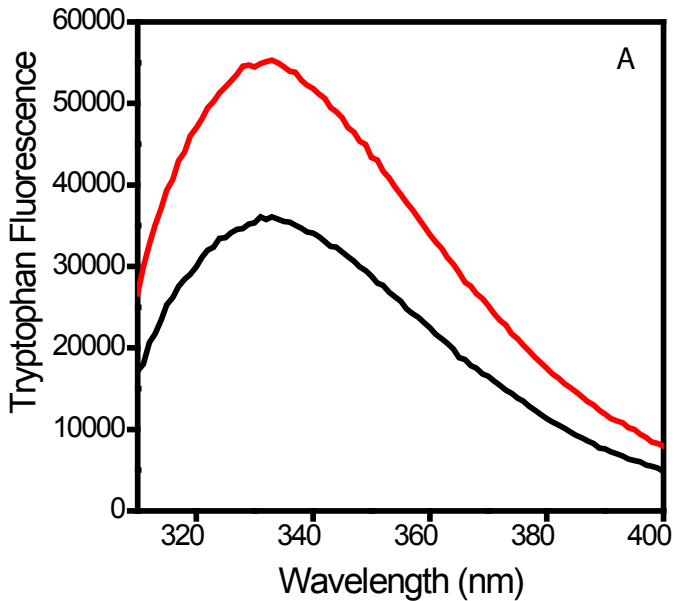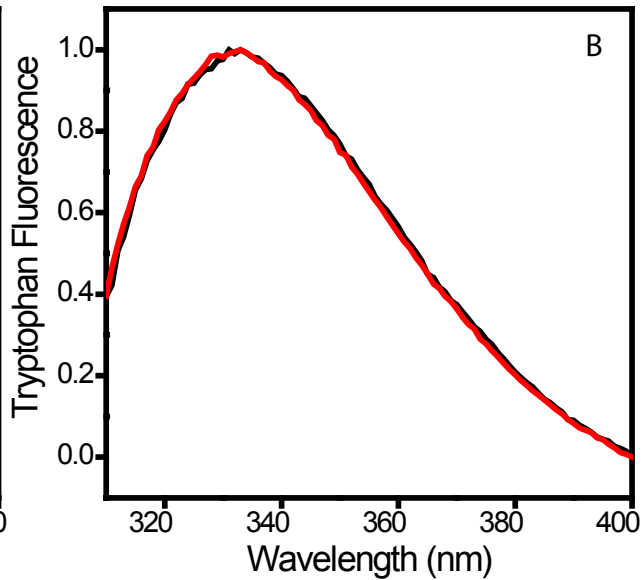

Supplement: Figure S5 — (A) Fluorescence spectra of Trp 78 of α-synuclein in the presence of POPG SUVs in absence (red) and in the presence (black) of 0.2 M potassium iodide (excited at 295 nm). (B) Normalized spectra showing no shift and no change in the spectral shape in the absence and in the presence of potassium iodide indicating that the observed shifts in the REES experiments are indeed due to slow water relaxation around Trp in the excited state. (PDF) [file pone.0083752.s005.pdf]

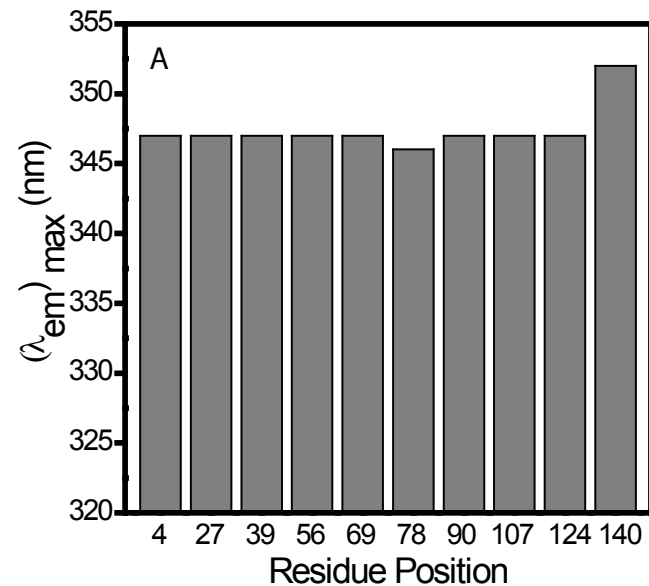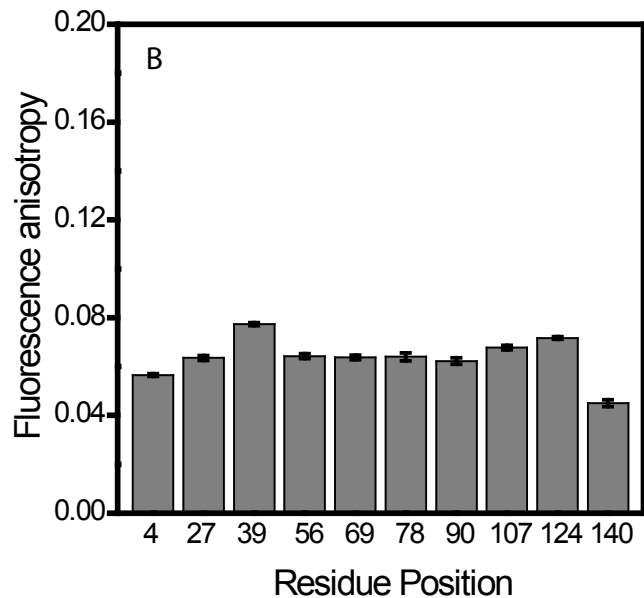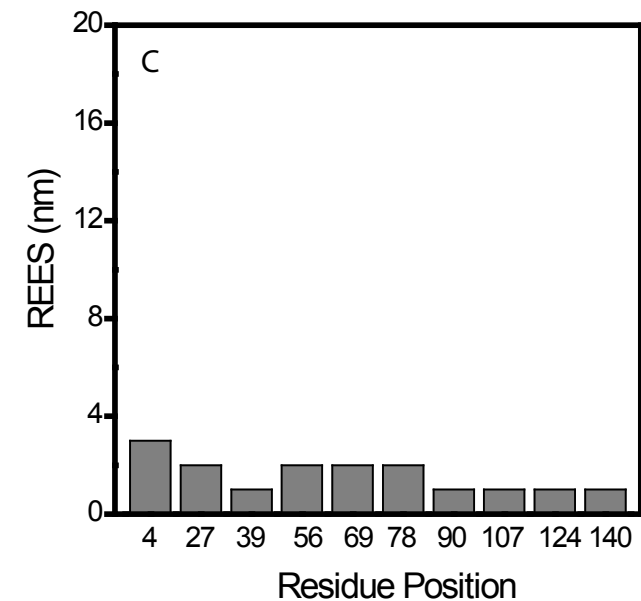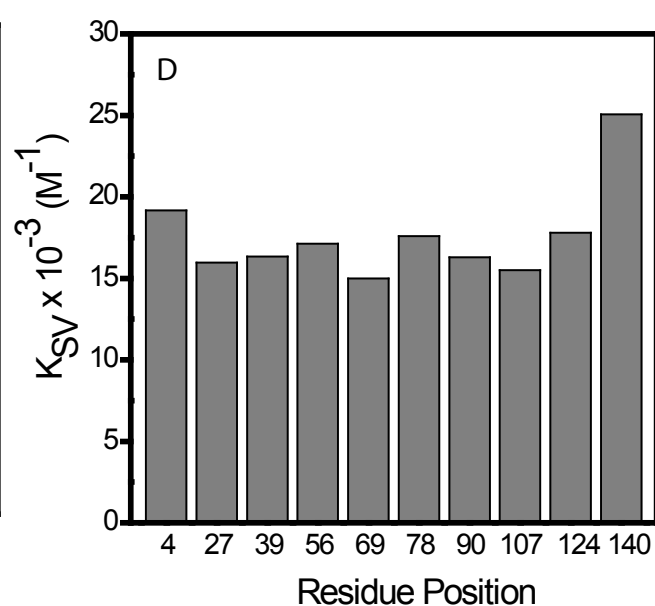

Supplement: Figure S6 — Different readouts of Trp variants in the presence of POPC SUVs. (A) Emission maxima (B) Fluorescence anisotropy (C) REES (D) Stern-Volmer constants. (PDF) [file pone.0083752.s006.pdf]
