# Supplementary material for: Structural and Dynamical Insights into the Membrane-Bound α-Synuclein
Source: PLoS One. 2013 Dec 20;8(12):e83752. doi: 10.1371/journal.pone.0083752 (PMC3869795; doi:10.1371/journal.pone.0083752)
Supplement: Table S1 — Primer sequences for site-directed mutagenesis. (PDF) [file pone.0083752.s007.pdf]

Table S1. Primer sequence for site-directed mutagenesis

Mutants	Primer	Sequence (5’→3’)	No. of Bases	T_m (°C)	% GC
A69W	Forward	5’GACAAATGTTGGAGGA***TGG***GTGGTGACG3’	28	60.1	53.6
A69W	Reverse	5’CACCCGTCACCAC***CCA***TCCTCCAAC3’	25	59.7	64
A27W	Forward	5’CCAAACAGGGTGTG***TGG***GAAGCAGCAGG3’	28	63.7	60.7
A27W	Reverse	5’GTCTTTCCTGCTGCTTC***CCA***CACACCCTG3’	29	62.5	58.6
A56W	Forward	5’GGTGTGGCAACAGTG***TGG***GAGAAGACC3’	27	58.6	59.3
A56W	Reverse	5’GCTCTTTGGTCTTCTC***CCA***CACTGTTGC3’	28	57.6	53.6
A90W	Forward	5’CAGGGAGCATTGCA***TGG***GCCACTGGC3’	26	65.2	65.4
A90W	Reverse	5’CAAAGCCAGTGGC***CCA***TGCAATGCTCC3’	27	64.4	59.3
A78W	Forward	5’GGTGTGACAGCAGTA***TGG***CAGAAGACAG3’	28	55.3	53.6
A78W	Reverse	5’CTCCACTGTCTTCTG***CCA***TACTGCTGTC3’	28	54.7	53.6
A107W	Forward	5’GCAAGAATGAAGAAGGA***TGG***CCACAGGAAG3’	30	61	50
A107W	Reverse	5’CAGAATTCCTTCCTGTGG***CCA***TCCTTCTTC3’	30	60.3	50
A124W	Forward	5’GGATCCTGACAATGAG***TGG***TATGAAATGCC3’	30	58.1	46.7
A124W	Reverse	5’CCTCAGAAGGCATTTCATA***CCA***CTCATTGTC3’	31	57.8	45.2
Y39W	Forward	5’GACAAAAGAGGGTGTTCTC***TGG***GTAGGCTCC3’	31	60.6	54.8
Y39W	Reverse	5’GGTTTTGGAGCCTAC***CCA***GAGAACACCC3’	28	59.2	57.1
F4W	Forward	5’ CATATGGATGTA***TGG***ATGAAAGGAC3’	25	45.4	40
F4W	Reverse	5’ GTCCTTTCAT***CCA***TACATCCATATG3’	25	45.4	40
A140W	Forward	5’GACTATGAACCTGAA***TGG***TAAGAAATATCTTT GC3’	34	55	35.3
A140W	Reverse	5’GAGCAAAGATATTTCTTA***CCA***TTCAGGTTCAT AG3’	34	55.1	35.3
